# High Prevalence of Gut Microbiota Colonization with Broad-Spectrum Cephalosporin Resistant *Enterobacteriaceae* in a Tunisian Intensive Care Unit

**DOI:** 10.3389/fmicb.2016.01859

**Published:** 2016-11-29

**Authors:** Elaa Maamar, Sana Ferjani, Ali Jendoubi, Samia Hammami, Zaineb Hamzaoui, Laure Mayonnove-Coulange, Mabrouka Saidani, Aouatef Kammoun, Amel Rehaiem, Salma Ghedira, Mohamed Houissa, Ilhem Boutiba-Ben Boubaker, Amine Slim, Veronique Dubois

**Affiliations:** ^1^Faculty of Medicine of Tunis - LR99ES09 Research Laboratory of Antimicrobial Resistance, University of Tunis El ManarTunis, Tunisia; ^2^Faculty of Sciences of Tunis, University of Tunis El ManarTunis, Tunisia; ^3^Intensive Care Unit, Charles Nicolle HospitalTunis, Tunisia; ^4^Faculty of Sciences of Gafsa, University of GafsaGafsa, Tunisia; ^5^University Bordeaux, Microbiologie Fondamentale et Pathogénicité, UMR5234Bordeaux, France; ^6^Laboratory of Microbiology, Charles Nicolle HospitalTunis, Tunisia

**Keywords:** cefotaxime resistance, gut colonization, ICU

## Abstract

Healthcare-associated infections due to cefotaxime-resistant (CTX-R) *Enterobacteriaceae* have become a major public health threat, especially in intensive care units (ICUs). Often acquired nosocomially, CTX-R *Enterobacteriaceae* can be introduced initially by patients at admission. This study aimed to determine the prevalence and genetic characteristics of CTX-R *Enterobacteriaceae*-intestinal carriage in ICU patients, to evaluate the rate of acquisition of these organisms during hospitalization, and to explore some of the associated risk factors for both carriage and acquisition. Between December 2014 and February 2015, the 63 patients admitted in the ICU of Charles Nicolle hospital were screened for rectal CTX-R *Enterobacteriaceae* colonization at admission and once weekly thereafter to identify acquisition. CTX-R *Enterobacteriaceae* fecal carriage rate was 20.63% (13/63) at admission. Among the 50 non-carriers, 35 were resampled during their hospitalization and the acquisition rate was 42.85% (15/35). Overall, 35 CTX-R *Enterobacteriaceae* isolates were collected from 28 patients (25 *Klebsiella pneumoniae*, seven *Escherichia coli*, and three *Enterobacter cloacae* strains). Seven patients were simultaneously colonized with two CTX-R *Enterobacteriaceae* isolates. CTX-M-15 was detected in most of the CTX-R *Enterobacteriaceae* isolates (30/35, 88.23%). Three strains co-produced CMY-4 and 22 strains were carbapenem-resistant and co-produced a carbapenemase [OXA-48 (*n* = 13) or NDM-1 (*n* = 6)]. Molecular typing of *K. pneumoniae* strains, revealed eight Pulsed field gel electrophoresis (PFGE) patterns and four sequence types (ST) [ST101, ST147, ST429, and ST336]. However, *E. coli* isolates were genetically unrelated and belonged to A (*n* = 2), B1 (*n* = 2) and B2 (*n* = 3) phylogenetic groups and to ST131 (two strains), ST572 (two strains), ST615 (one strain) and ST617 (one strain). Five colonized patients were infected by CTX-R *Enterobacteriaceae* (four with the same strain identified from their rectal swab and one with a different strain). Whether imported or acquired during the stay in the ICU, colonization by CTX-R *Enterobacteriaceae* is a major risk factor for the occurrence of serious nosocomial infections. Their systematic screening in fecal carriage is mandatory to prevent the spread of these multidrug resistant bacteria.

## Introduction

Gastrointestinal colonization with multidrug resistant bacteria (MDR; resistant to three or more classes of antibiotics), including cefotaxime-resistant (CTX-R) *Enterobacteriaceae*, may be associated with subsequent clinical infection and this constitutes a reservoir for transmission that may remain unidentified in hospitals which do not implement active surveillance testing. Many factors are associated with CTX-R *Enterobacteriaceae* fecal carriage including antibiotic exposure, malignancy, non-surgical invasive procedures, prolonged hospital stay, admission to intensive care units (ICU), and sharing a room with known carriers (Thiébaut et al., 2012). A recent study emphasized on the importance of identifying individuals carrying antimicrobial resistant bacteria in both patient and healthy populations (Ben Sallem et al., 2014). An increase in the proportion of carriers in the community increases the risk that other individuals will also become carriers via human-to-human transmission. In addition, the admission into hospital of carriers of MDR bacteria increases the risk of other hospitalized patient to acquire the fecal carriage or also contract an infection with these MDR bacteria (Razazi et al., 2012).

In Tunisia, investigations of fecal carriage of MDR bacteria are rare (Ben Sallem et al., 2012; Tarchouna et al., 2014; Sana et al., in press). The present prospective study aimed to determine the prevalence of CTX-R *Enterobacteriaceae*-intestinal carriage in patients newly admitted at the ICU of Charles Nicolle hospital, to evaluate the rate of acquisition of these organisms during their ensuing hospitalization, and to explore some of the associated risk factors for both carriage and acquisition.

## Patients and methods

### Design of the study

A prospective study including all patients with fecal colonization or infection due to CTX-R *Enterobacteriaceae* was conducted in the ICU of the Charles Nicolle Hospital of Tunis (Tunisia) from December 2014 to February 2015. The unit had 12 beds located in private rooms. For each patient, the demographic characteristics, medical history, and reason for hospitalization were recorded. A single rectal swab specimen was collected from each patient at admission. For original non-carriers, follow-up cultures were performed at weekly intervals until hospital discharge or positive detection. Imported carriers were defined as patients found to be colonized with CTX-R *Enterobacteriaceae* at admission. The acquisition rate was defined as the number of patients who were not colonized at admission but become colonized afterwards, divided by the number of patients not colonized at admission. Medical record review was done by one of the investigators, after clearance of the ward departmental heads.

### Sample processing and microbial study

Each rectal swab was inoculated onto desoxycolate lactatose agar (Biokar diagnostics) supplemented with cefotaxime (2 mg/L). Each enterobacteria colonies which were judged to differ in morphology (size, shape, consistency, and color) were selected from each sample and identified using the API 20E system (Biomerieux).

Antimicrobial susceptibility testing to 18 antibiotics (amoxicillin, amoxicillin-clavulanic acid, ceftazidime, cefotaxime, aztreonam, cefoxitin, imipenem, ertapenem, gentamicin, amikacin, tobramycin, nalidixic acid, ciprofloxacin, trimethoprim-sulfamethoxazole, tetracyclin, minocyclin, fosfomycin, and chloramphenicol) was determined by the agar disk diffusion method on Mueller-Hinton (MH) agar plates (Bio-Rad) according to the CA-SFM guidelines (http://www.sfm-microbiologie.org/).

Isolates were screened for extended-spectrum beta-lactamases (ESBL)-phenotype by double-disk synergy test (DDST) with cefotaxime, ceftazidime, and amoxicillin-clavulanic acid disks. CTX-R *Enterobacteriaceae* isolates showing a negative-ESBL-phenotype with resistance to amoxicillin-clavulanic acid and to cefoxitin were classified as AmpC producers. For isolates showing decreased susceptibility to carbapenems, a modified Hodge test (MHT) was conducted as previously described (Poirel et al., 2011a).

### Risk factor analysis

To investigate the risk factors associated with fecal carriage within groups (imported and acquired carriers), carriers were compared with non-carriers in terms of exposure to the different variables studied. Carrier patients were defined as those with one rectal swab test-positive result for CTX-R *Enterobacteriaceae*. Carriage detected more than 72 h after ICU admission was considered ICU-acquired CTX-R *Enterobacteriaceae*. The period between admission and acquisition of CTX-R *Enterobacteriaceae* carriage was defined as the surveillance period. Demographic data, characteristics at admission (patient's location prior to admission, co-morbidities), antibiotic exposure during ICU stay, and outcome were collected for CTX-R *Enterobacteriaceae* carriers and non-carriers. Univariate analysis of discrete variables was performed using the two-sided Pearson's chi-squared and Fisher's exact tests (a *P* < 0.05 was considered statistically significant).

### Detection and characterization of β-lactamase genes

Detection and characterization of *bla* genes were performed by PCRs and sequencing. Isolates showing positive DDST were screened for *bla*_CTX−M_, *bla*_SHV_, *bla*_TEM_, and *bla*_GES_ (Saladin et al., 2002), while those with AmpC-phenotype were screened for plasmid-mediated AmpC-β-lactamases genes (*bla*_MOX_, *bla*_CIT_, *bla*_DHA_, *bla*_ACC_, *bla*_EBC_, and *bla*_FOX_; Pérez-Pérez and Hanson, 2002). Isolates with reduced susceptibility to carbapenems and positive MHT were screened for *bla*_OXA-48_, *bla*_KPC_, *bla*_NDM_, and *bla*_GES_ genes (Poirel et al., 2011a).

### Detection of associated plasmid-mediated quinolone resistance (PMQR) genes

The presence of plasmid-mediated genes associated with quinolones resistance (*qnrA, qnrB*, and *qnrS*) was determined by multiplex PCR and sequencing (Cattoir et al., 2007).

### Genetic environments

The genetic environments of *bla*_CTX-*M*-15_, *bla*_OXA-48_, and *bla*_NDM-1_ genes were determined by PCR mapping using specific primers as described previously (Eckert et al., 2006; Poirel et al., 2011b, 2012).

### Transfer of resistance determinants

Genetic support was studied by matting-out assays for all strains. Clinical strains were used as donors. For rifampin-susceptible isolates, a rifampin-, and nalidixic acid-resistant *E. coli* K12 strain was used as recipient, while for rifampin-resistant and sodium azide-susceptible isolates, a sodium azide resistant *E. coli* C600 was used as recipient. Transconjugants were selected on MH agar plates containing sodium azide (100 mg/L) or rifampicin (250 mg/L) and cefotaxime (2 mg/L). Transconjugants resistance profiles were tested by disk diffusion method and PCR.

### Molecular typing of isolates

*E. coli* isolates were assigned to the phylogenetic groups A, B1, B2, or D using a PCR strategy with specific primers for *chuA, yjaA*, and *TspE4*.C2 determinants as previously described (Clermont et al., 2000).

The epidemiological relationship between the different isolates was determined by pulsed-field gel electrophoresis (PFGE) of *XbaI*-digested genomic DNA of all recovered isolates as previously described (Sáenz et al., 2004). Patterns were visually compared and analyzed according to previously reported criteria (Tenover et al., 1995; Sáenz et al., 2004). Although, when identical PFGE patterns of strains (*K. pneumoniae* and *E. coli*) were observed, representative strains were selected for multilocus sequence typing (MLST), performed as previously described (Sáenz et al., 2004). All the amplicons were sequenced and compared with the sequences deposited in the MLST database (http://bigsdb.web.pasteur.fr/), to know the specific allele combination and the sequence type (ST).

### Virulence factors

*E. coli* isolates were screened for the presence of 18 virulence genes [*fimH, afa/draBC, sfa/focDE, papG* (allele I, II, and III), *cnf1, sat, hlyA, iutA, iroN, fyuA, iha, kpsM II, ompT, traT*, and *usp*] using multiplex PCRs. Isolates were defined as extra-intestinal pathogenic *E. coli* (ExPEC) if positive for two of the following genes or gene sets: *papA* and/or *papC* (P fimbriae), *sfa/focDE* (S and F1C fimbriae), *afa/draBC* (Dr-binding adhesins), *kpsM II* (group 2 capsule), and *iutA* (aerobactin siderophore system; Johnson et al., 2003).

*K. pneumoniae* isolates were screened for the presence of nine virulence genes (*ycfM, mrkD, entB, ybtS, kfu, iroN, magA, allS*, and *rmpA*) using multiplex PCRs (Lafeuille et al., 2013).

The virulence score was calculated as the sum of all virulence factors (VF) for which the isolate tested positive.

## Results

### Study population and prevalence of CTX-R *Enterobacteriaceae* carriage

**Figure 1:**
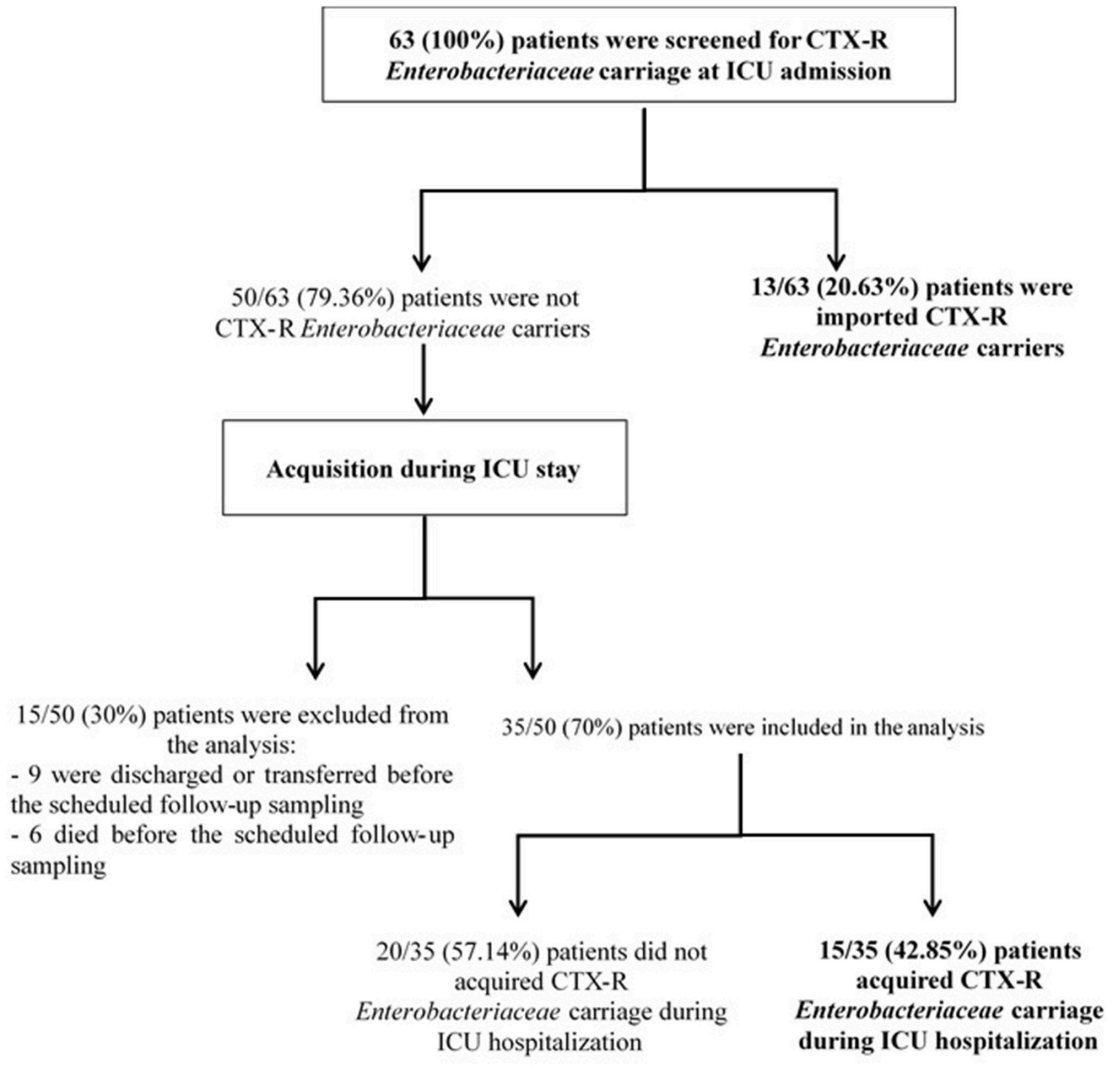
**Flow chart of the patients**.

During the study period, 63 patients were admitted to the ICU of the Charles Nicolle Hospital. The male-to-female ratio was 1.25; the median age was 54 years (range, 7–87 years; Table 1). The median length of ICU stay was 6 days (2–136 days). Most patients were admitted for medical reasons (69.8%, 44/63) and transferred from different wards of our hospital (80.95%, 51/63). All patients received mechanical ventilation and had venous and/or arterial devices. Twenty-eight patients (44.44%) were screened only once, while 35 (55.55%) patients had at least two swabs (range, 2–5 swabs). CTX-R *Enterobacteriaceae* prevalence at admission was 20.63% (13/63). Of the 50 non-carriers, follow-up rectal swabs were realized for only 35 patients, resulting in a CTX-R *Enterobacteriaceae* acquisition rate of 42.85% (15/35). The 15 remaining patients were excluded (9 were discharged or transferred and six died before the scheduled follow-up sampling; Figure 1). Twenty-one patients harbored a single CTX-R *Enterobacteriaceae* species and seven harbored two different CTX-R *Enterobacteriaceae* species. The median time between admission in ICU and acquisition of carriage was 6 days (range, 3–15 days).

**Table 1:** **Characteristics of patients with cefotaxime-resistant ***Enterobacteriaceae*** in fecal carriage at ICU admission and during ICU hospitalization**.

**Variables**	**Imported CTX-R *Enterobacteriaceae* carriers (*n* = 13)**	**Non-imported CTX-R *Enterobacteriaceae* carriers (*n* = 50)**	**ICU-acquired CTX-R *Enterobacteriaceae* carriers (*n* = 15)**	**Non-ICU-acquired CTX-R *Enterobacteriaceae* carriers (*n* = 20)**
Age, median (range)	60 (11–78)	54 (7–79)	53 (29–87)	47 (7–79)
≤ 15 years	1	3	0	2
16<<59 years	5	29	9	13
≥60 years	7	18	7	4
Gender (Male) *n* (%)	7 (53.84)	12 (63)	10 (66.6)	12 (60)
Reason of ICU admission n (%)				
Medical reason for ICU admission	7 (53.84)	37 (74)	12 (80)	13 (65)
Surgery before ICU admission	6 (46.15)	13 (26)	4 (26.6)	6 (30)
Polytraumatism	2 (15.38)	8 (16)	–	–
Antibiotic use before ICU admission *n* (%)	7 (53.84)	12 (24)	3 (20)	6 (30)
3rd cephalosporin generation	2	8	8 (53.3)	6 (30)
Carbapenem	1	2	5 (33.3)	8 (40)
Amoxicillin-clavulanic acid	5	4	8 (53.3)	10 (50)
Fluoroquinolone	0	3	10 (66.6)	7 (35)
Aminoside	3	3	6 (40)	2 (10)
Glycopeptide	–	–	3 (20)	6 (30)^*^
Hospital days before ICU admission, median (range)	5 (2–28)	3 (1–17)^*^	–	–
≤ 72 h	3	28	–	–
>72 h	10	22	–	–
**COMORBIDITIES,** ***n***				
Diabetes	3	13	4	5
HTA	3	10	2	6
Infection at ICU admission, *n* (%)	5 (38.46)	3 (6) ^*^	2 (12.5)	0 (0)
Infection with CTX-R *Enterobacteriaceae* during ICU hospitalization, *n* (%)	–	–	4 (25)	2 (10.52)
**ORIGIN**				
Charles Nicolle hospital wards	9	42	13	17
Emergency unit	2	15	5	6
Cardiology	0	4	1	3
Surgical units	2	2	2	2
Gynecology	0	5	1	1
Neurology	0	2	0	2
Orthopedic	1	4	2	2
Urology	1	4	1	0
Gastrology	1	0	–	–
Medicine	1	1	–	–
Maxillo-facial	0	1	–	–
ORL	0	1	1	0
Pediatric	1	2	0	1
Pneumology	0	1	–	–
Private hospital	4	8	3	2
**OUTCOMES,** ***n*** **(%)**				
Death	2 (15.38)	22 (44) ^*^	8 (53)	8 (40)
Discharge or transferred	11 (84.61)	28 (56)	7 (46.6)	12 (60)
Hospital days before ICU admission, median (range)	–	–	3.5 (2–14)	5 (1–15)
≤ 72 h, *n*	–	–	8	9
>72 h, *n*	–	–	8	10
ICU days before acquisition, median (range)	–	–	5 (3–15)	10 (4–25)
≤ 72 h, *n*	–	–	2	0
>72 h, *n*	–	–	14	19

* *0.05 ≤ P ≤ 0.007; –, not applicable; CTX-R Enterobacteriaceae, cefotaxime-resistant Enterobacteriaceae*.

### Risk factors for CTX-R *Enterobacteriaceae* carriage

In univariate analysis, significant risk factors for CTX-R *Enterobacteriaceae* carriage on admission were prior antibiotic use (*P* = 0.03) and infection at admission (*P* = 0.007; Table 1). In addition, CTX-R *Enterobacteriaceae* fecal colonization at admission was significantly associated with patients' outcomes (*P* = 0.05). However, risk factors such as age, gender, location prior ICU admission, comorbidities such as diabetes and hypertension, previous hospitalization, and duration did not show any significant correlation with carriage of CTX-R *Enterobacteriaceae* (*P* > 0.05). No significant risk factor for CTX-R *Enterobacteriaceae* hospital acquisition in univariate analysis was observed (Table 1). All patients (carriers and non-carriers) underwent invasive procedures including intravenous or urinary catheterization during hospitalization.

### Bacterial species identification and distribution of resistance genotypes of *CTX-R enterobacteriaceae* isolates

In all, 35 CTX-R *Enterobacteriaceae* strains were isolated from 28 patients. Ten *K. pneumoniae*, six *E. coli*, and two *Enterobacter cloacae* isolates were identified at admission, while 15 *K. pneumoniae*, one *E. coli*, and one *E. cloacae* isolates were acquired later. The acquisition rates of fecal carriage of cefotaxime-resistant *K. pneumoniae, E. coli* and *E. cloacae* were 42.85% (15/35), 2.85% (1/35), and 2.85% (1/35), respectively. All isolates, except one (AmpC-phenotype), were ESBL producers (*n* = 34). Among the ESBL producers, three showed the combination of the both phenotypes, ESBL and AmpC. Associated resistances were as follows: cefoxitin (*n* = 24), ertapenem (*n* = 22), imipenem (*n* = 19), gentamicin (*n* = 27), tobramycin (*n* = 29), amikacin (*n* = 11), nalidixic acid (*n* = 32), ciprofloxacin (*n* = 31), tetracyclin (*n* = 29), chloramphenicol (*n* = 34), and trimethoprim-sulfamethoxazole (*n* = 29; Table 2). Among the carbapenem-resistant strains, 16 showed a positive MHT.

**Table 2:**
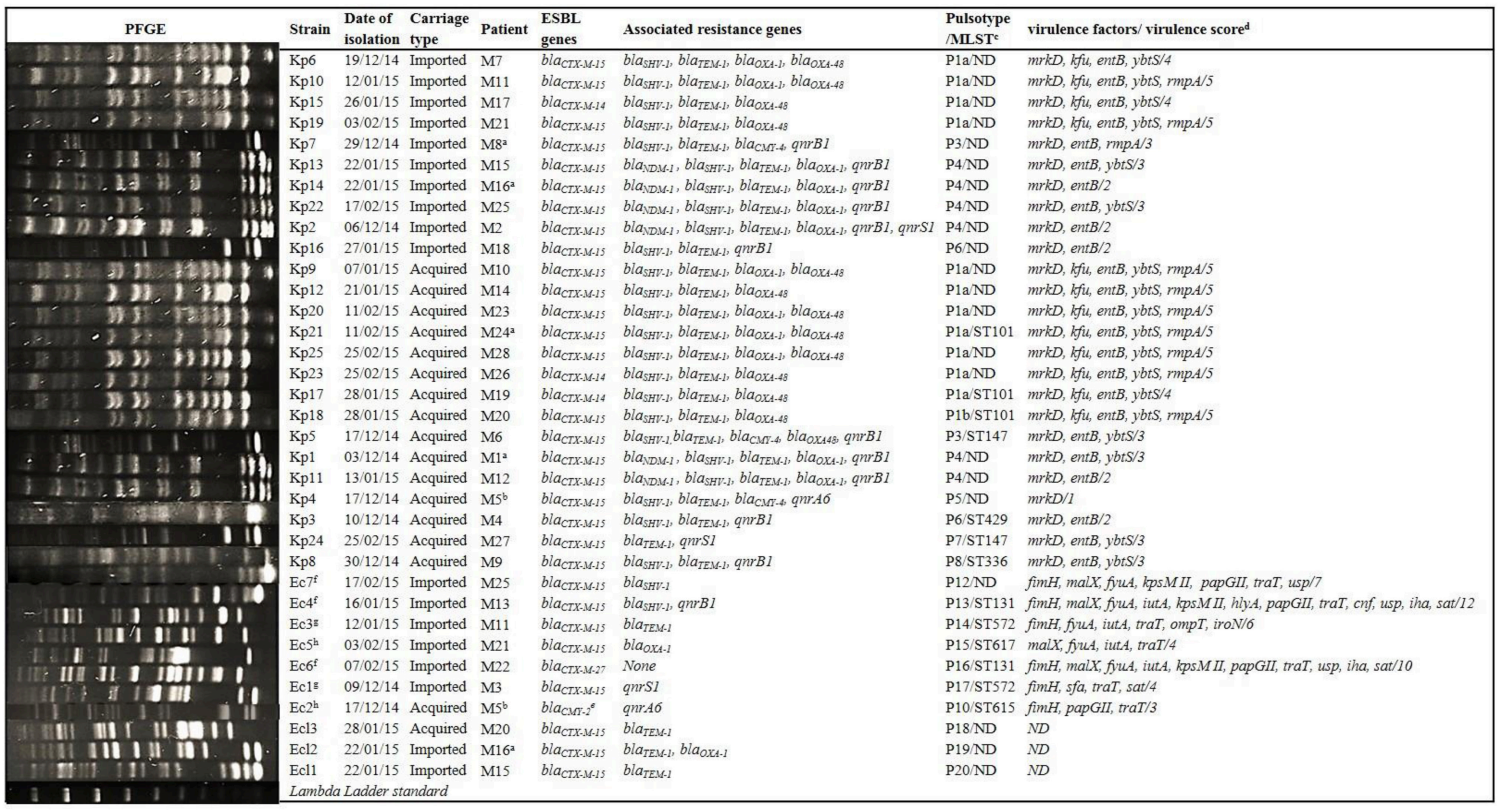
*****XbaI***-PFGE profile and characteristics of cefotaxime-resistant ***Enterobacteriaceae*** strains collected in intestinal flora of ICU-patients**.

*Ecl, Enterobacter cloacae; Kp, Klebsiella pneumonia; Ec, E. coli; ND, not determined; ESBL, extended-spectrum-beta-lactamase; PFGE, pulsed-field gel electrophoresis; MLST, multi locus sequence typing; ST, sequence type*.

*^a^Patient infected by the same strain recovered in his rectal swab*.

*^b^Patient infected and colonized by two different strains*.

*^c^MLST was performed only for selected strains*.

*^d^Virulence score was calculated as the sum of all virulence genes for which the isolates tested positive*.

*^e^Plasmidic AmpC producing-strain (CMY-2 type) but none ESBL producer*.

*^f^E. coli belonged to phylogroups B2*.

*^g^E. coli belonged to phylogroups B1*.

*^h^E. coli belonged to phylogroups A*.

### β-lactamase genes detection and PMQR genes

ESBL genes were identified as *bla*_CTX-*M*-15_ (*n* = 30), *bla*_CTX-*M*-14_ (*n* = 3), and *bla*_*CTX*−*M*−27_ (*n* = 1). The strain with the AmpC-phenotype (Ec2) harbored *bla*_CMY-2_. Among the *bla*_CTX-*M*-15_-producers, three co-produced *bla*_CMY-4_, six *bla*_NDM-1_, and 10 *bla*_OXA-48_. All *bla*_CTX-*M*-14_-producers harbored *bla*_OXA-48_. In addition, *bla*_TEM-1_, *bla*_SHV-1_, and *bla*_OXA-1_ genes were concomitantly present in 29, 26, and 14 isolates, respectively (Table 2). *qnrB1, qnrS1*, and *qnrA6* were detected in 13, 3, and 2 strains, respectively.

### Transfer of resistance

Fifteen over thirty (50%) CTX-M-15 ESBL were successfully transferable by conjugation. However, transfer of CTX-M-14, CTX-M-27, and CMY-2 enzymes were not successful, despite three separate attempts. *bla*_OXA-1_, *bla*_TEM-1_, and *bla*_NDM-1_ genes were co-transferred in 13, 12, and 5 cases, respectively. Depending on the strain, other resistances were co-transferred, mostly gentamicin (12/13; 92.3%), tetracyclin (6/13; 46.15%) and rarely trimethoprim-sulfamethoxazole (2/8; 25%; Table 3). In addition, *bla*_CMY-4_ and *bla*_OXA-48_ genes were transferable alone in three and seven strains, respectively and co-transferred in one strain (TCKp5).

**Table 3:** **Characteristics of cefotaxime-resistant ***Enterobacteriaceae*** strains collected in intestinal flora of ICU-patients and their transconjugants**.

**Strain**	**Resistance profile to non-beta-lactams**	**ESBL genes**	**Associated *bla* genes**
Kp6	GEN, AMK, TOB, NET, MNO, TET, NAL, CIP, SXT	*bla*_CTX-*M*-15_	*bla*_SHV-1_, *bla*_TEM-1_, *bla*_OXA-1_, *bla*_OXA-48_
TCKp6	GEN, TOB, NET, AMK	*bla*_CTX-*M*-15_	*bla*_TEM-1_, *bla*_OXA-1_
Kp10	GEN, AMK, TOB, NET, MNO, TET, NAL, CIP, SXT	*bla*_CTX-*M*-15_	*bla*_SHV-1_, *bla*_TEM-1_, *bla*_OXA-1_, *bla*_OXA-48_
TCKp10 (1)	GEN, TOB, NET, AMK, TET	*bla*_CTX-*M*-15_	*bla*_TEM-1_, *bla*_OXA-1_
TCKp10 (2)	None	None	*bla*_OXA-48_
Kp15^b^	GEN, AMK, TOB, NET, MNO, TET, NAL, CIP, CHL, SXT	*bla*_CTX-*M*-14_	*bla*_SHV-1_, *bla*_TEM-1_, *bla*_OXA-48_
Kp19^b^	GEN, AMK, TOB, NET, MNO, TET, NAL, CIP, CHL	*bla*_CTX-*M*-15_	*bla*_SHV-1_, *bla*_TEM-1_, *bla*_OXA-48_
Kp7	GEN, TOB, NET, MNO, TET, NAL, CIP, CHL, SXT	*bla*_CTX-*M*-15_	*bla*_SHV-1_, *bla*_TEM-1_, *bla*_CMY-4_
TCKp7	TET, CHL, SXT	None	*bla*_CMY-4_
Kp13	GEN, TOB, NET, MNO, TET, NAL, CIP, SXT	*bla*_CTX-*M*-15_	*bla*_*NDM*−1_, *bla*_SHV-1_, *bla*_TEM-1_, *bla*_OXA-1_
TCKp13	GEN, TOB, NET	*bla*_CTX-*M*-15_	*bla*_NDM-1_, *bla*_TEM-1_, *bla*_OXA-1_
Kp14^b^	GEN, TOB, NET, MNO, TET, NAL, CHL, SXT	*bla*_CTX-*M*-15_	*bla*_NDM-1_, *bla*_SHV-1_, *bla*_TEM-1_, *bla*_OXA-1_
Kp22	GEN, TOB, NET, MNO, TET, CIP, SXT	*bla*_CTX-*M*-15_	*bla*_NDM-1_, *bla*_SHV-1_, *bla*_TEM-1_, *bla*_OXA-1_
TCKp22	GEN, TOB	*bla*_CTX-*M*-15_	*bla*_NDM-1_, *bla*_TEM-1_, *bla*_OXA-1_
Kp2	GEN, TOB, NET, MNO, TET, NAL, CIP, FOS, SXT	*bla*_CTX-*M*-15_	*bla*_NDM-1_, *bla*_SHV-1_, *bla*_TEM-1_, *bla*_OXA-1_
TCKp2	GEN, TOB	*bla*_CTX-*M*-15_	*bla*_NDM-1_, *bla*_TEM-1_, *bla*_OXA-1_
Kp16^b^	GEN, NET, MNO, NAL, CIP, SXT	*bla*_CTX-*M*-15_	*bla*_SHV-1_, *bla*_TEM-1_
Kp9	GEN, AMK, TOB, NET, MNO, TET, NAL, CIP, FOS, SXT	*bla*_CTX-*M*-15_	*bla*_SHV-1_, *bla*_TEM-1_, *bla*_OXA-1_, *bla*_OXA-48_
TCKp9 (1)	TET, TOB, NET, AMK, FOS	*bla*_CTX-*M*-15_	*bla*_TEM-1_, *bla*_OXA-1_
TCKp9 (2)	FOS	None	*bla*_OXA-48_
Kp12	GEN, TOB, NET, MNO, TET, CIP, CHL, SXT	*bla*_CTX-*M*-15_	*bla*_SHV-1_, *bla*_TEM-1_, *bla*_OXA-48_
TCKp12	None	None	*bla*_OXA-48_
Kp20	GEN, AMK, TOB, NET, NAL, CIP, FOS	*bla*_CTX-*M*-15_	*bla*_SHV-1_, *bla*_TEM-1_, *bla*_OXA-1_, *bla*_OXA-48_
TCKp20 (1)	TET, GEN, TOB, NET	*bla*_CTX-*M*-15_	*bla*_TEM-1_, *bla*_OXA-1_
TCKp20 (2)	None	None	*bla*_OXA-48_
Kp21	GEN, AMK, TOB, NET, MNO, TET, NAL, CIP, CHL	*bla*_CTX-*M*-15_	*bla*_SHV-1_, *bla*_TEM-1_, *bla*_OXA-1_, *bla*_OXA-48_
TCKp21 (1)	GEN, TOB, NET, AMK, FOS	*bla*_CTX-*M*-15_	*bla*_TEM-1_, *bla*_OXA-1_
TCKp21 (2)	FOS	None	*bla*_OXA-48_
Kp25	GEN, TOB, AMK, NET, CIP, NAL, TET	*bla*_CTX-*M*-15_	*bla*_SHV-1_, *bla*_TEM-1_, *bla*_OXA-1_, *bla*_OXA-48_
TCKp25	TET, GEN, TOB, AMK, FOS	*bla*_CTX-*M*-15_	*bla*_TEM-1_, *bla*_OXA-1_
Kp23^b^	GEN, AMK, TOB, NET, MNO, TET, NAL, CIP, FOS, CHL, SXT	*bla*_CTX-*M*-14_	*bla*_SHV-1_, *bla*_TEM-1_, *bla*_OXA-48_
Kp17	GEN, AMK, TOB, NET, TET, NAL, CIP, FOS, CHL, SXT	*bla*_CTX-*M*-14_	*bla*_SHV-1_, *bla*_TEM-1_, *bla*_OXA-48_
TCKp17	None	None	*bla*_OXA-48_
Kp18^b^	GEN, TOB, NET, TET, NAL, SXT	*bla*_CTX-*M*-15_	*bla*_SHV-1_, *bla*_TEM-1_, *bla*_OXA-48_
Kp5	GEN, TOB, NET, MNO, TET, NAL, CIP, SXT	*bla*_CTX-*M*-15_	*bla*_*SHV*−1,_ *bla*_TEM-1_, *bla*_CMY-4_, *bla*_*OXA*48_
TCKp5	TET	None	*bla*_*OXA*−48,_*bla*_CMY-4_
Kp1	GEN, TOB, NET, MNO, TET, NAL, CIP, FOS, SXT	*bla*_CTX-*M*-15_	*bla*_NDM-1_, *bla*_SHV-1_, *bla*_TEM-1_, *bla*_OXA-1_
TCKp1	GEN, TOB	*bla*_CTX-*M*-15_	*bla*_NDM-1_, *bla*_TEM-1_, *bla*_OXA-1_
Kp11	GEN, TOB, NET, MNO, TET, NAL, CIP, SXT	*bla*_CTX-*M*-15_	*bla*_NDM-1_, *bla*_SHV-1_, *bla*_TEM-1_, *bla*_OXA-1_
TCKp11	GEN, TOB	*bla*_CTX-*M*-15_	*bla*_NDM-1_, *bla*_TEM-1_, *bla*_OXA-1_
Kp4	AMK, TOB, MNO, TET, NAL, CIP, SXT	*bla*_CTX-*M*-15_	*bla*_SHV-1_, *bla*_TEM-1_, *bla*_CMY-4_
TCKp4	TET, SXT	None	*bla*_CMY-4_
Kp3^b^	GEN, TOB, NET, NAL, CIP, SXT	*bla*_CTX-*M*-15_	*bla*_SHV-1_, *bla*_TEM-1_
Kp24^b^	TOB, NET, TET, NAL, CIP, SXT	*bla*_CTX-*M*-15_	*bla*_TEM-1_
Kp8^b^	TOB, NET, MNO, TET, NAL, CIP, SXT	*bla*_CTX-*M*-15_	*bla*_SHV-1_, *bla*_TEM-1_
Ec7	None	*bla*_CTX-*M*-15_	*bla*_SHV-1_
TCEc7	None	*bla*_CTX-*M*-15_	None
Ec4^b^	GEN, TOB, NET, MNO, TET, NAL, CIP, SXT	*bla*_CTX-*M*-15_	*bla*_SHV-1_
Ec3	MNO, TET, CHL, SXT	*bla*_CTX-*M*-15_	*bla*_TEM-1_
TCEc3	None	*bla*_CTX-*M*-15_	None
Ec5	GEN, TOB, MNO, TET, NAL, CIP, SXT	*bla*_CTX-*M*-15_	*bla*_OXA-1_
TCEc5	GEN, TOB, TET, SXT	*bla*_CTX-*M*-15_	*bla*_OXA-1_
Ec6^b^	TET, NAL, CIP, NOR, SXT	*bla*_*CTX*−*M*−27_	None
Ec1^b^	NAL, CIP	*bla*_CTX-*M*-15_	None
Ec2^b^	MNO, TET, NAL, CIP, CHL, SXT	*bla*_CMY−2_^a^	None
Ecl3^b^	GEN, TOB, MNO, NAL, CIP, NOR, CHL, SXT	*bla*_CTX-*M*-15_	*bla*_TEM-1_
Ecl2^b^	GEN, TOB, NET, MNO, TET, NAL, CIP, CHL, SXT	*bla*_CTX-*M*-15_	*bla*_TEM-1_, *bla*_OXA-1_
Ecl1	GEN, TOB, NET, MNO, TET, NAL, CIP, CHL, SXT	*bla*_CTX-*M*-15_	*bla*_TEM-1_
TCEcl1	TET, GEN, TOB, CHL, SXT	*bla*_CTX-*M*-15_	*bla*_TEM-1_, *bla*_OXA-1_

*Ecl, Enterobacter cloacae; Kp, Klebsiella pneumoniae; Ec, E. coli; NAL, nalidixic acid; CIP, ciprofloxacin; SXT, trimethoprim-sulfamethoxazole; TET, tetracyclin; MNO, minocyclin; GEN, gentamicin; TOB, tobramycin; AMK, amikacin; NET, netilmicin, CHL, chloramphenicol; FOS, fosfomycin; ND, not determined; ESBL, extended-spectrum-beta-lactamase; TC, Transconjugant*.

a *plasmidic AmpC producing-strain (CMY-2 type) but none ESBL producer*.

b *resistance transfer was not obtained*.

### Molecular typing of isolates

Among the 25 non-duplicated *K. pneumoniae* strains (isolated from 25 patients), 21 were clustered in four PFGE patterns labeled P1a (11 strains), P3 (2 strains), P4 (6 strains), and P6 (2 strains). Singletons were labeled P1b, P5, P7, and P8 (Table 2). These PFGE patterns corresponded to ST101 for PFGE patterns P1a and P1b, to ST147 for P3 and P7 patterns and to ST429 and ST336, for P6 and P8 PFGE patterns, respectively (Table 2). In contrast, PFGE analysis showed heterogeneous clones among *E. coli* (labeled P10, 12, 13, 14, 15, 16, and 17) and *E. cloacae* (labeled P18, 19, and 20) isolates (Table 2).

In addition, phylogenetic analysis revealed that *E. coli* isolates belonged to the following groups: A (*n* = 2), B1 (*n* = 2), and B2 (*n* = 3); and the following sequence types associated phylogenetic groups were detected: ST131-B2 (2 strains), ST572-B1 (2 strains), ST615-A (one strain), and ST617-A (one strain).

### Virulence genotyping of cefotaxime-resistant *E. coli* and *K. pneumoniae*

Of the 18 VFs sought, *fimH* virulence gene was detected in all cefotaxime-resistant *E. coli* isolates (except in Ec5), *papGII* in 4, *iutA* in 4, *malX* in 4, *iroN* in 1 isolate. VF scores were in the range of 3–12 (median, 6). *E. coli* isolate Ec4, typed as ST131-B2, contained 12 VFs (*fimH, malX, fyuA, iutA, KpsM II, hlyA, papGII, traT, cnf, usp, iha, and sat*). Nevertheless, *papGI, papGIII*, or *afa*/*draBC* virulence genes were not detected in any isolate (Table 2).

The *mrkD* gene was detected in all *K. pneumoniae* isolates. The *entB, ybtS, kfu*, and *rmpA* genes were detected in 24, 18, 12, and 10 isolates, respectively (Table 2). VF scores were in the range of 1–5 (median, 3).

### Genetic environment of *bla*_CTX-M-15_, *bla*_NDM-1_, and *bla*_OXA-48_

*ISEcp1* were identified upstream of *bla*_CTX-*M*-15_ in 26 strains. The organization *ISAba125-bla*_NDM-1_-*ble*_MBL_ and *LysR-bla*_*OXA*−48_*-Tnp1999* were identified as genetic structures surrounding *bla*_NDM-1_ in all strains and *bla*_OXA-48_ in 11 strains, respectively.

### Nosocomial infections

During the study, seven cases of ICU-acquired infections by CTX-R *Enterobacteriaceae* occurred among the 63 hospitalized patients. The cause of infection was septicemia (*n* = 3), pulmonary infection (*n* = 2), catheter related infection (*n* = 1) and intra-abdominal infection (*n* = 1). The incidence rate of ICU-acquired CTX-R *Enterobacteriaceae* infections was estimated at 11.1% during the study period. Five of the seven infected patients were also CTX-R *Enterobacteriaceae* carriers; four of them were infected with their carriage strain (patients M1, M8, M16, and M24) and the remaining patient (patient M5) was infected with a different strain. For this patient (M5), infection preceded the CTX-R *Enterobacteriaceae* carriage. For the two infected patients and non-carriers of CTX-R *Enterobacteriaceae*, they were infected with strains P1 and P8 pulsotypes. Thus, all acquired infections cases were caused by *K. pneumoniae* strains which belonged to the same ST clones that colonized ICU hospitalized patients in the same period (Table 4). All ICU-acquired infections were caused by CTX-M-15 producing *K. pneumoniae* strains (*n* = 7). Five of them co-produced a carbapenemase [OXA-48 (*n* = 3) and NDM-1 (*n* = 2)] and one co-produced CMY-4 (Table 2).

**Table 4:** **Comparison of ST types bacteria: colonization ***vs***. infection**.

**PFGE/MLST**	**Colonization (imported/acquired)**	**Infection**
P1/ST101 (*n* = 14)	11(4/7)	3
P3/ST147 (*n* = 4)	2(1/1)	1
P8/ST336 (*n* = 1)	1(0/1)	1
P4/ND (*n* = 6)	6(4/0)	2

*PFGE, pulsed-field gel electrophoresis; MLST, multi locus sequence typing; ST, sequence type; ND, not determined*.

The median length of ICU stay before the occurrence of ICU-acquired infections caused by CTX-R *Enterobacteriaceae* was 12 days (range, 3–20 days). The median time elapsed between the demonstration of the CTX-R *Enterobacteriaceae*-carriage and the diagnosis of the ICU-acquired infection was 5.5 days (range, 3–12 days).

## Discussion

CTX-R *Enterobacteriaceae* are considered as a major cause of morbidity and mortality worldwide. They are becoming prevalent causal agents of nosocomial infections, especially in ICUs (Bonten and Weinstein, 1996; Razazi et al., 2012). Epidemiologic features of circulation of these organisms have been described by several authors, who have identified risk factors for microbiota colonization and infection and applied molecular tracing as a tool for assessing transmission pathways, cross-transmission burden and addressing drug-resistance control strategies (Razazi et al., 2012; Thiébaut et al., 2012; Ko et al., 2013; Kim et al., 2014).

The main finding from this 3-month study is the high rate of fecal carriage of CTX-R *Enterobacteriaceae* at ICU admission (20.63%), with *K. pneumoniae* representing the most common CTX-R *Enterobacteriaceae* species recovered (76.92%) especially for the acquired-carriage; which is in accordance with previous studies (Thiébaut et al., 2012; Tarchouna et al., 2014). These MDR bacteria were probably selected during hospitalization preceding ICU transfer; however, a community origin cannot be eliminated. Imported CTX-R *Enterobacteriaceae* carriers are transferred from different wards of Charles Nicolle Hospital or from private hospitals, which demonstrate dissemination in the entire hospital and implying inter-institutional or even regional transmission of these pathogens. In accordance with other reports (Razazi et al., 2012; Thiébaut et al., 2012), we found that duration of hospitalization before ICU admission was significantly associated with imported fecal carriage (*P* = 0.05). In addition, a high percentage of imported carriers had received antibiotics before their ICU admission, which has certainly played an important role in the selection of CTX-R *Enterobacteriaceae*. However, no statistically significant relationship was found (*P* = 0.08). A weakness of the present study was that we were unable to collect data about patients in relation with their prior hospitalization and antibiotic use in the 3 months preceded their admission.

The admission screening is mandatory for its importance in population assessment of the full reservoir of organisms and the respect of proper practice guidelines like contact isolation and sterile barrier precautions are advocated.

In our study, CTX-R *Enterobacteriaceae* acquisition rate was alarming (42.85%), higher than that reported in France and Korea (Razazi et al., 2012; Kim et al., 2014). The median time of stay in our ICU for acquisition of CTX-R *Enterobacteriaceae* (5 days) was slightly lower to those reported in China (7 days) and France (9 days; Razazi et al., 2012; Ma et al., 2015). Almost all patients (carriers and non-carriers) received antibiotics and all of them underwent invasive procedures including intravenous or urinary catheterization during hospitalization. Thus, no specific risk factors to acquired CTX-R *Enterobacteriaceae* among gut microbiota have been identified, in univariate analysis, except the use of glycopeptides (*P* = 0.05).

However, in contrast with the very high frequency of CTX-R *Enterobacteriaceae* colonization, infection occurred only in 11.11% of patients, accordingly with other studies. Asymptomatically colonized patients may act as a reservoir for persistence of CTX-R *Enterobacteriaceae* in the hospital setting and this may provide further rationale for routine surveillance screening in high-risk populations. Notably, four of the patients who had an ICU-acquired infection caused by CTX-R *Enterobacteriaceae* were found to have been previously colonized in an average of 5.75 days (2–10 days) before infection with the same strain. Therefore, a major risk factor for nosocomial infection is prior colonization, as previously reported (Bonten and Weinstein, 1996).

In Tunisia, the fecal carriage of ESBL-producing *Enterobacteriaceae* (ESBL-E) was reported in three previous studies: one investigated children hospitalized in a pediatric unit, one concerning healthy adults and another about healthy children (Ben Sallem et al., 2012; Tarchouna et al., 2014; Sana et al., in press). All these findings raise the question of asymptomatic carriage of this pathogen not only in the hospital settings but also in the community, which enhances the spread of resistance genes by transmission from human-to-human, patients-to-others and medical staff-to-patients or contamination of the environment in our country.

Multiple investigators reported that most colonizing ESBL-E isolates were *K. pneumoniae* in high-risk patients and associated with hospital-onset infections, whereas *E. coli* was frequently isolated in healthy individuals and was the predominant cause of community-onset infections (Ben Sallem et al., 2012; Ko et al., 2013; Tarchouna et al., 2014).

Our finding showed the diversity of β-lactamase genes including *bla*_CTX-*M*-15_, *bla*_CTX-*M*-14_, *bla*_*CTX*−*M*−27_, and *bla*_CMY-2_. *bla*_CTX-*M*-15_, the most dominant gene detected among our strain collection, is the most prevalent ESBL gene in human clinical isolates worldwide (Bonnet, 2004; Chouchani et al., 2011). It is worth-mentioning that CTX-M β-lactamase genes are widely known to be carried on a plasmid linked to mobile genetic elements that are utilized as vehicles for resistance genes horizontal movement, in addition to carry on resistance to other antibiotics like aminoglycosides and fluoroquinolones. Multidrug resistance profiles involving non-β-lactamase antibiotics in ESBL-producing isolates may also contribute to the increase in colonization pressure. Fluoroquinolone resistance is becoming a common feature rather than an exception in ESBL-producing isolates (Rodriguez-Martínez et al., 2011). Recent studies have demonstrated co-transfer of *qnr* determinants on ESBL-producing plasmids conferring resistance to nalidixic acid with reduced susceptibility to fluoroquinolones.

In conclusion, imported or acquired CTX-R *Enterobacteriaceae* colonization was found with high prevalence. The majority of colonizing CTX-R *Enterobacteriaceae* isolates was MDR strains co-carrying diverse resistance determinants. Molecular epidemiologic analysis showed the circulation of clonally related strains (mainly *K. pneumoniae*) showing that cross-transmission play a major role on their acquisition.

Whether imported or acquired during the stay in ICU, intestinal colonization by CTX-R *Enterobacteriaceae* is a major risk factor for the occurrence of serious nosocomial infections with limitations in the therapeutic options available for their treatment. Thus, their systematic screening in fecal carriage, with strict adherence to patient hygiene and infection control practices are mandatory to prevent the spread of these MDR bacteria.

## Author contributions

EM, IB-BB, SG, MH and AS designed the study. EM, SF, SH, ZH and LMC performed lab experiments and participated in the analysis of the data. EM and SF performed bioinformatic and statistical analysis. AJ, VD, MS, AK and AR provided expertise, participated in the analysis of data, and in the revision of the manuscript. EM, SF, and IB-BB performed the analysis of data and wrote the manuscript. All authors read and approved the final version of the manuscript.

## Funding

This work was supported by the Tunisian Ministry of Higher Education and Scientific Research.

### Conflict of interest statement

The authors declare that the research was conducted in the absence of any commercial or financial relationships that could be construed as a potential conflict of interest.
